# Automated quantitative MRI volumetry reports support diagnostic interpretation in dementia: a multi-rater, clinical accuracy study

**DOI:** 10.1007/s00330-020-07455-8

**Published:** 2021-01-15

**Authors:** Hugh G. Pemberton, Olivia Goodkin, Ferran Prados, Ravi K. Das, Sjoerd B. Vos, James Moggridge, William Coath, Elizabeth Gordon, Ryan Barrett, Anne Schmitt, Hefina Whiteley-Jones, Christian Burd, Mike P. Wattjes, Sven Haller, Meike W. Vernooij, Lorna Harper, Nick C. Fox, Ross W. Paterson, Jonathan M. Schott, Sotirios Bisdas, Mark White, Sebastien Ourselin, John S. Thornton, Tarek A. Yousry, M. Jorge Cardoso, Frederik Barkhof, for the Alzheimer’s Disease Neuroimaging Initiative

**Affiliations:** 1 Centre for Medical Image Computing (CMIC), Department of Medical Physics and Bioengineering, University College London, London, UK; 2 Neuroradiological Academic Unit, UCL Queen Square Institute of Neurology, University College London, London, UK; 3 Dementia Research Centre, UCL Queen Square Institute of Neurology, University College London, London, UK; 4 Universitat Oberta de Catalunya, Barcelona, Spain; 5 Clinical, Educational and Health Psychology, University College London, London, UK; 6 Lysholm Department of Neuroradiology, National Hospital for Neurology and Neurosurgery, UCLH NHS Foundation Trust, London, UK; 7 Department of Neuroradiology, Brighton and Sussex University Hospitals, Brighton, UK; 8 Guy’s and St Thomas’ NHS Foundation Trust, London, UK; 9 Department of Diagnostic and Interventional Neuroradiology, Hannover Medical School, Hannover, Germany; 10 Department of Surgical Sciences, Radiology, Uppsala University, Uppsala, Sweden; 11 Department of Radiology and Nuclear Medicine, Erasmus MC University Medical Center, Rotterdam, the Netherlands; 12 Department of Epidemiology, Erasmus MC University Medical Center, Rotterdam, the Netherlands; 13 Digital Services, University College London Hospital, London, UK; 14 School of Biomedical Engineering and Imaging Sciences, King’s College London, London, UK; 15 Radiology & Nuclear Medicine, VU University Medical Center, Amsterdam, the Netherlands

**Keywords:** Alzheimer’s disease, Frontotemporal dementia, Radiologists, Magnetic resonance imaging, Atrophy

## Abstract

**Objectives:**

We examined whether providing a quantitative report (QReport) of regional brain volumes improves radiologists’ accuracy and confidence in detecting volume loss, and in differentiating Alzheimer’s disease (AD) and frontotemporal dementia (FTD), compared with visual assessment alone.

**Methods:**

Our forced-choice multi-rater clinical accuracy study used MRI from 16 AD patients, 14 FTD patients, and 15 healthy controls; age range 52–81. Our QReport was presented to raters with regional grey matter volumes plotted as percentiles against data from a normative population (*n* = 461). Nine raters with varying radiological experience (3 each: consultants, registrars, ‘non-clinical image analysts’) assessed each case twice (with and without the QReport). Raters were blinded to clinical and demographic information; they classified scans as ‘normal’ or ‘abnormal’ and if ‘abnormal’ as ‘AD’ or ‘FTD’.

**Results:**

The QReport improved sensitivity for detecting volume loss and AD across all raters combined (*p* = 0.015* and *p* = 0.002*, respectively). Only the consultant group’s accuracy increased significantly when using the QReport (*p =* 0.02*)*.* Overall, raters’ agreement (Cohen’s *κ*) with the ‘gold standard’ was not significantly affected by the QReport; only the consultant group improved significantly (*κ*_*s*_ 0.41➔0.55, *p =* 0.04*). Cronbach’s alpha for interrater agreement improved from 0.886 to 0.925, corresponding to an improvement from ‘good’ to ‘excellent’.

**Conclusion:**

Our QReport referencing single-subject results to normative data alongside visual assessment improved sensitivity, accuracy, and interrater agreement for detecting volume loss. The QReport was most effective in the consultants, suggesting that experience is needed to fully benefit from the additional information provided by quantitative analyses.

**Key Points:**

*• The use of quantitative report alongside routine visual MRI assessment improves sensitivity and accuracy for detecting volume loss and AD vs visual assessment alone.*

*• Consultant neuroradiologists’ assessment accuracy and agreement (kappa scores) significantly improved with the use of quantitative atrophy reports.*

*• First multi-rater radiological clinical evaluation of visual quantitative MRI atrophy report for use as a diagnostic aid in dementia.*

**Supplementary Information:**

The online version contains supplementary material available at 10.1007/s00330-020-07455-8.

## Introduction

Brain magnetic resonance imaging (MRI) is regularly used in diagnosing dementia as it visualises the structural changes caused by neurodegeneration [1, 2]. In particular, MRI is key in defining subtle differences between healthy and pathological cerebral volume loss and between dementia subtypes [3]. These changes can be challenging to identify in both research and clinical settings, as evidenced by moderate interrater variability [4].

Several visual rating scales have been developed to enable reproducible semiquantitative assessment of volume loss [5–10]. They have been shown to reduce interrater variability to such a degree that they are used in clinical trials [11–13]**.** However, these scales have a subjective element and their application relies heavily on the prior experience of the radiologist using them. Furthermore, they have poor sensitivity to subtle or prodromal changes and have ceiling and/or floor effects [4]. These shortcomings can be addressed by using total and regional volume quantification, which has been used as an outcome measure in research studies and clinical trials [11, 14, 15]. It has been suggested that quantification can also improve diagnostic accuracy, reliability, confidence, and efficiency by providing region-specific volumetric differences between single subjects and an age-matched normative population [16–21]. The clinical introduction of volume quantification is however predicated upon technical and clinical validation, as well as compliance with mandatory governance regulations [22–24].

**Fig. 1:**
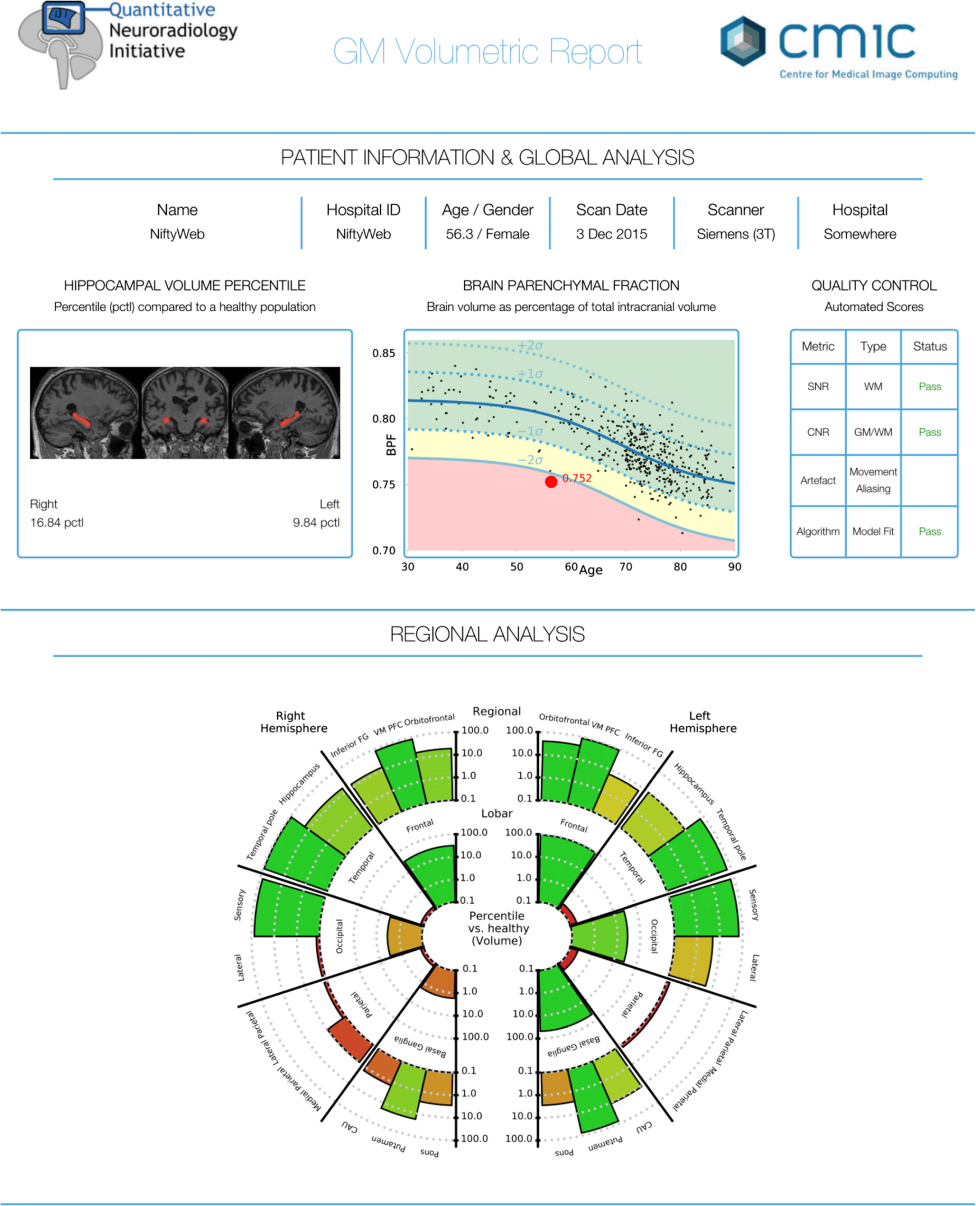
Quantitative report (QReport) of an AD patient displaying demographics, hippocampal volume percentiles, and single-subject brain parenchymal fraction (red dot) plotted against a normative dementia-free population. Quality control metrics and a ‘rose plot’ representation of GM volume percentiles split by brain lobe and relevant sub-regions. The rose plot is on a log scale and uses a traffic light colour-coding system (green to red meaning high to low percentile) to display the individual’s volume percentiles in the context of a healthy population. *Abbreviations*: BPF, brain parenchymal fraction; SNR, signal-to-noise ratio; CNR, contrast-to-noise ratio; GM, grey matter; WM, white matter; CAU, caudate

We have developed a pipeline that automatically generates a novel and clinically usable quantitative report (QReport—Fig. 1). The segmentation algorithm we have used is Geodesic Information Flows (GIF), which is part of the in-house software NiftySeg (http://niftyweb.cs.ucl.ac.uk/program.php?p=GIF) [25]. Our pipeline integrates and displays a patient’s demographic information, MRI quality control metrics, GIF’s hippocampal segmentation, and volumetric results contextualised against a normative population. The QReport generates a ‘rose plot’ representation, which displays complex 3D data in a visually simple and easily interpreted 2D format [26]. Evaluation of most commercial reports has been limited to CE and FDA approval; this study aims to fulfil step 4 in the Quantitative Neuroradiology Initiative (QNI) six-step framework by evaluating how the QReport affects clinical accuracy [24].

In this study, we assessed the effect of our QReport across two diagnostic steps and three neuroradiological levels of experience. We hypothesised (1) that the use of our QReport will decrease interrater variability whilst increasing diagnostic specificity, sensitivity, accuracy, and confidence (a) for determining the presence of volume loss and (b) for determining the differential diagnosis of AD or FTD; and (2) that the QReport’s effect will be identifiable across the three experience levels.

## Methods

### Patient dataset

We established a test set of MRI scans from 45 subjects scanned locally, using three different 3-T MRI systems (see supplementary material for acquisition parameters). Fifteen ‘control subjects’ were referred to our specialist clinic on memory concerns but deemed to fall within normal ranges upon neurological, cerebrospinal fluid (CSF) and imaging assessment. MMSE scores have been included as a marker of cognitive performance (see Table 1).

Thirty patients were diagnosed with either AD (*n* = 16, beta-amyloid 1–42 < 550 pg/mL and tau:amyloid ratio > 1) or FTD (*n* = 14), based on clinical evaluation and CSF markers. MMSE scores and disease duration are provided in Table 1. All data were acquired under ethical approval by the Queen Square ethics committee: 13 LO 0005 and 12 LO 1504.

### Reference dataset

The normative healthy control data were derived from the Alzheimer’s Disease Neuroimaging Initiative (ADNI) database (*n* = 382; age range 56–90 years) (adni.loni.usc.edu) augmented by the Track-HD study cohort [27] to include younger controls (*n* = 79; age range = 30–65 years) thereby covering a clinically appropriate age range. The total normative population was *n* = 461 (51.4% female), and the mean age was 70.09 years, SD = 12.05. Subject data in the ‘reference dataset’ were acquired under the ethical agreements in place for ADNI and TRACK-HD studies.

### Quantification and display of grey matter volumes

Whole brain, grey matter, and relevant regional volumes were estimated for all participants using Geodesic Information Flows (GIF). GIF provides fully automated multi-atlas segmentation and global and region-specific volumetry of T1-weighted scans. It has been validated in manual segmentation studies both in dementia and other neurodegenerative disorders [25, 28–30]. This is especially relevant for the comparison of morphologically different subjects, as examined in this study [25, 31]. We developed an automated pipeline that presents data in a clinically usable report format (Fig. 1) displaying non-identifying demographics, hippocampal volume percentiles, and brain parenchymal fraction plotted against normative population data. Regional brain volumes were expressed as percentile estimates against a Gaussian distribution approximation of healthy control grey matter volumes, after regressing out age, gender, and total intracranial volume. We used a variant of a generalised logistic function to predict the values of our observational normative database as a continuous variable. This allowed us to compute the cumulative distribution function of measured values with respect to the normative population. Data were displayed in a visually simple and intuitive ‘rose plot’ format.

### Study design

**Fig. 2:**
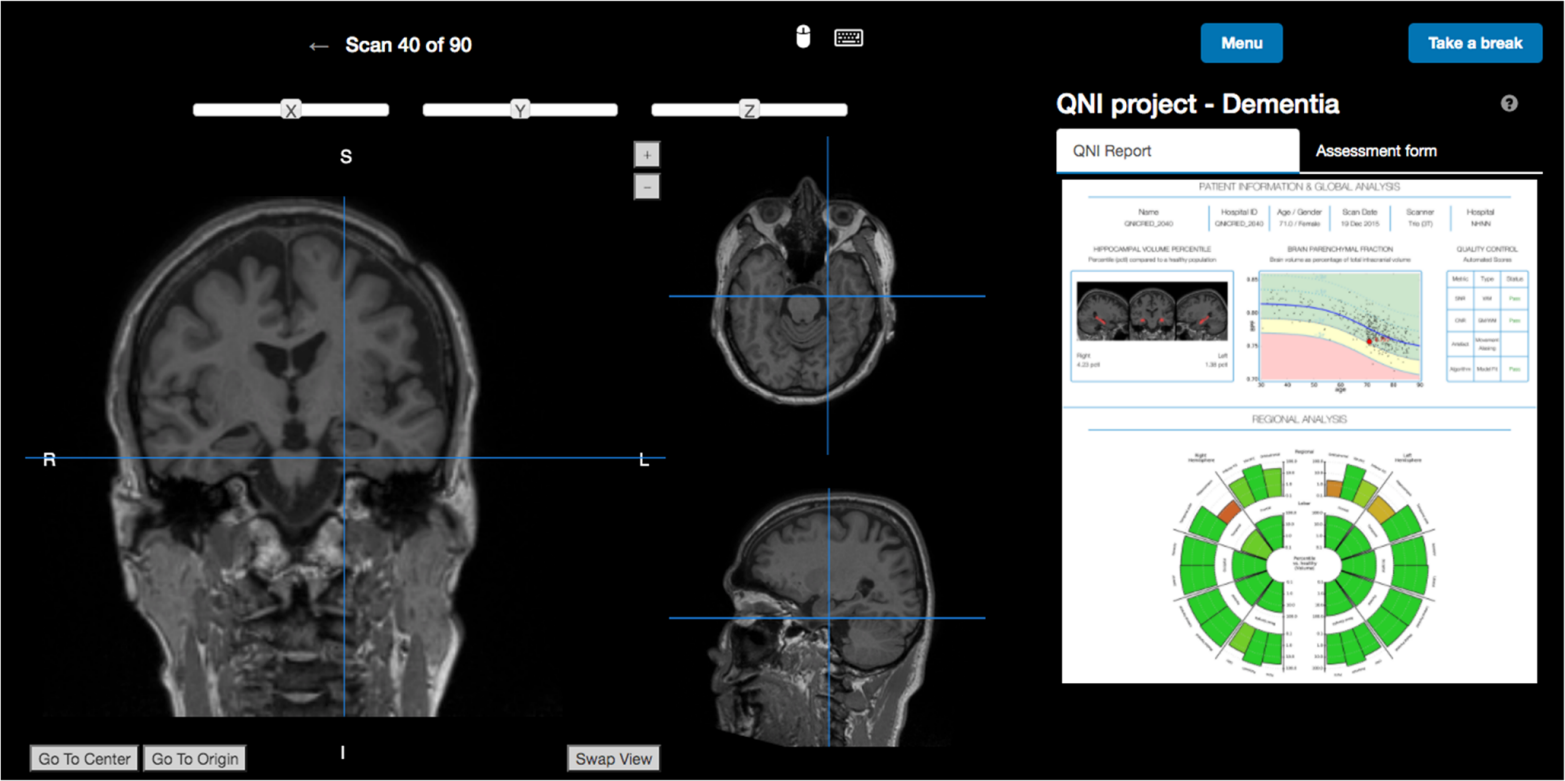
Screenshot from the Quantitative Neuroradiology Initiative (QNI) study website (http://qni.cs.ucl.ac.uk) showing the image viewer for a case with the QReport available. QReports were fully interactive and zoomable via the website

Three groups of raters participated in this study: consultant neuroradiologists; neuroradiology specialty registrars; and non-clinical image analysts. Raters were invited from multiple centres, ensuring a broad representation of training and experience. Raters were blinded to all clinical and demographic information except for age and gender. We designed a website platform (Fig. 2) to facilitate remote participation. The website included thorough instructions (see supplementary material) followed by 45 scans displayed once with and once without the QReport available. In order to mitigate against systematic learning or anchoring effects, scans were automatically randomised and delivered to raters in a unique order per rater through our rating website. The task consisted of 90 evaluation ‘episodes’ in total.

At each ‘episode’, raters were prompted to give their assessment, stating (1.a) whether the scan was ‘normal’ or ‘abnormal’ in terms of volume loss for age; (1.b) degree of confidence on a scale of 1 (very uncertain) to 5 (very confident); (2.a) if the scan was rated abnormal, to select AD or FTD; and (2.b) their confidence level for this differential diagnosis (1–5 scale). Raters completed the exercise over a period of 2 months; ratings were collected through the web platform and subsequently analysed.

### Statistical analysis

We explored the effects of QReport availability on the accuracy of (1) identifying volume loss (normal versus abnormal) and (2) differential diagnosis of AD versus FTD. Key signal-detection indices were calculated using the following ratings: (a) *correctly* defined as ‘abnormal’ (‘*true positive*’ for AD/FTD), and ‘normal’ (‘*true negative*’ for healthy controls) and (b) *incorrectly* defined as ‘abnormal’ (‘*false positive’* for healthy controls) and ‘normal’ (‘*false negative’* for patients). Using these metrics, diagnostic sensitivity, specificity, and accuracy were calculated and expressed as percentages as follows:

$$ Accuracy=\frac{True\ Positives+ True\  Ne\mathrm{g} atives}{True\ Positives+ True\ Negatives+ False\ Positives+ False\ Negatives}\times \kern0.5em 100 $$

$$ Sensitivity=\frac{True\ Positives}{True\ Positives+ False\ Negatives}\times 100 $$

$$ Specificity=\frac{True\ Negatives}{True\ Negatives+ False\ Positives}\times 100 $$

Subsequently, counts of correctly and incorrectly diagnosed scans with and without the QReport available were analysed with the McNemar test. Paired *t* tests were used to assess mean diagnostic accuracy, specificity, and sensitivity across the two conditions (QReport present vs absent). Cohen’s kappa was calculated to assess agreement between raters’ evaluations and confirmed diagnosis while accounting for ‘chance’ agreement. To further assess the effect of the QReport’s availability on consistency and reliability among raters, Cronbach’s alpha and intraclass correlation coefficients were calculated.

Confidence ratings (QReport vs no QReport) were calculated as a grand mean per rater and for each ‘true’ disease type (normal, AD, FTD) and assessed with paired *t* tests. In exploratory analyses rating, normal vs abnormal, we hypothesised that the effects of the QReport on confidence and diagnostic ratings could vary depending on whether the rated scans were normal or abnormal and whether the raters correctly classified the scans and the experience level of the raters. A four-way mixed ANOVA, including all factors (QReport × normality × correctness × experience level), allowed us to assess how these factors interact*.*

All statistical analyses were performed with SPSS version 24.

## Results

### Assessment accuracy

#### Volume loss: normal vs abnormal

For all raters combined, the availability of the QReport significantly improved the diagnostic sensitivity (p = 0.015*), without changing the specificity or accuracy. However, for accuracy, a beneficial medium effect size (0.53) was observed. Of the 3 rating groups, only the consultant group’s accuracy improved significantly, from 71 to 80% (p = 0.02*) (Table 2).

**Table 1:** Characteristics of the test subject data set. Mean age was matched across subjects, mean Abeta 1–42 was reduced and mean Tau was raised for AD subjects relative to controls. Mean MMSE was significantly lower for AD (*p* < 0.001) and FTD (*p* = 0.03) when compared with ‘controls’. Mean disease duration (time from first reported symptom to MRI) in y is also shown

	Controls (*n* = 15)	AD (*n* = 16)	FTD (*n* = 14)	Total (*n* = 45)
Age in years, mean (SD)	60 (8.7)	61.7 (6.6)	59.9 (7.3)	60.6 (7.4)
Gender male:female	4:11	9:7	11:3	24:21
Mean Abeta 1–42 (pg/mL)	878.8	393.3	747.7	–
Mean Tau (pg/mL)	373.3	855.2	302.6	–
MMSE, mean (SD)	26.9 (4)	20.5 (6.4)	22 (9.1)	–
Disease duration in years, mean (SD)	–	2.7 (1.6)	3.5 (2.4)	–

#### AD vs FTD

The presence of the QReport significantly improved sensitivity for AD in the image analysts (p = 0.01*) and for all raters combined (p = 0.002*) (Table 3). There were no significant changes in diagnosing FTD (Table 4). In absolute terms, the number of correct diagnoses of AD and FTD increased with the report by 6.9% and 5.6%, respectively, with a medium effect size for AD, but these changes were not significant.

**Table 2:** Sensitivity, specificity, and accuracy for normal vs abnormal rating across all experience levels, both with and without the quantitative report

Metric	Experience level	Without report mean (SD)	With report mean (SD)	*p* value	*d* effect size
Sensitivity	Consultant	68.9% (5)	80% (10)	0.13	1.4
	Registrar	75.5% (8.4)	81.1% (1.9)	0.3	0.8
	Image analyst	70% (25.1)	85.5% (10.1)	0.23	0.9
	All groups combined	71.5% (13.8)	82.2% (7.6)	0.015*	1.03
Specificity	Consultant	75.6% (3.8)	80% (13.3)	0.52	0.43
	Registrar	82.2% (10.1)	68.8% (25.2)	0.37	− 0.6
	Image analyst	77.7% (3.8)	68.9% (13.8)	0.45	− 0.52
	All groups combined	78.5% (6.4)	72.3% (16.8)	0.3	− 0.37
Accuracy	Consultant	71.1% (2.2)	80% (2.2)	0.02*	4
	Registrar	77.7% (3.8)	77% (9.2)	0.87	− 0.1
	Image analyst	72.6% (17.9)	80% (2.2)	0.5	0.46
	All groups combined	73.8% (9.5)	79% (5.1)	0.15	0.53

*Statistically significant at < 0.05

**Table 3:** Sensitivity, specificity, and accuracy for AD vs normal rating across all experience levels, and percentage of correct assessments for AD, both with and without the quantitative report

Metric	experience level	Without report mean (SD)	With report mean (SD)	*p* value	*d* effect size
Sensitivity	Consultant	61.3% (12.9)	75.8% (17)	0.05	0.96
	Registrar	79.3% (12.7)	83.9% (4.7)	0.42	0.48
	Image analyst	61.7% (45.1)	71.1% (44.4)	0.01*	0.22
	All groups combined	67.4% (25.8)	76.9% (24.5)	0.002*	0.37
Specificity	Consultant	79.6% (8)	82% (14.6)	0.73	0.2
	Registrar	83.1% (7.5)	78. 1% (7.8)	0.46	− 0.65
	Image analyst	86.6% (4.2)	77.9% (12.9)	0.31	− 0.9
	All groups combined	83.1% (6.6)	79.3% (10.7)	0.3	0.42
Accuracy	Consultant	70.7% (3.2)	79.2% (10.9)	0.07	1.05
	Registrar	80.3% (2.3)	78.9% (7.4)	0.76	− 0.25
	Image analyst	75.8% (16.8)	77.9% (4.2)	0.84	0.17
	All groups combined	75.5% (9.6)	78.7% (4.3)	0.38	0.43
Correct AD diagnoses		58.1% (3.4)	65% (4.1)	0.128	0.56

*Statistically significant at < 0.05

### Assessment confidence

For rating normal vs abnormal, using a four-way mixed ANOVA (*QReport × normality × correctness × experience level*), we found a *normality × correctness × QReport interaction*, indicating significantly increased confidence when *incorrectly* rating abnormal scans with the QReport (i.e. false-positive judgement). These findings represent a statistically significant difference (*p* = 0.02 and *F*(1,8) = 7.918), with a small effect size (*η*^2^_p_ = 0.497), which did not vary across experience level groups. Raters were also significantly more confident:

1. With the QReport than without, regardless of correctness [*F*(1,8) = 6.64, *p* = 0.03, *η*^2^_p_ = 0.453]
2. When correctly rating, regardless of QReport use [*F*(1,8) = 112.43, *p* = < 0.01, *η*^2^_p_ = 0.934]
3. When rating abnormal rather than normal scans, regardless of QReport use [*F*(1,8) = 21.68, *p* = < 0.01, *η*^2^_p_ = 0.73]

There were no other significant effects on confidence when using the QReport.

### Agreement between raters and gold standard—Kappa scores

Cohen’s kappa scores for each rater when detecting volume loss (abnormal) are detailed in Table 5, and for differentiating between AD or FTD in Table 6. For both assessments, only the consultant group’s kappa scores increased significantly when using the QReport (p = 0.038* and p = 0.04*, respectively).

**Table 4:** Sensitivity, specificity, and accuracy for FTD vs normal rating across all experience levels, and percentage of correct assessments for FTD, both with and without the quantitative report

Metric	Experience level	Without report mean (SD)	With report mean (SD)	*p* value	*d* effect size
Sensitivity	Consultant	57.3% (4.1)	57.2% (6.2)	0.93	− 0.01
	Registrar	36.5% (7.8)	35.2% (23.8)	0.94	− 0.07
	Image analyst	46.9% (24.9)	58.3% (20.2)	0.1	0.5
	All groups combined	46.9% (16)	50.3% (19.5)	0.52	0.19
Specificity	Consultant	89.2% (9.4)	95% (6.5)	0.19	0.71
	Registrar	91.1% (9.7)	77.7% (32.7)	0.42	− 0.55
	Image analyst	75.5% (28.9)	85.5% (14.5)	0.46	0.43
	All groups combined	85.2% (17.6)	86.1% (19.7)	0.89	0.04
Accuracy	Consultant	73.6% (4.9)	75.9% (3.6)	0.09	0.53
	Registrar	70.5% (11)	65.2% (22.8)	0.52	0.29
	Image analyst	69.1% (15.9)	72.6% (14.3)	0.41	0.23
	All groups combined	71.1% (10.2)	71.2% (14.4)	0.95	0.01
Correct FTD diagnoses		38.6% (2.2)	44.2% (2.7)	0.367	0.31

**Table 5:** Kappa scores for normal/abnormal assessments across all experience levels, both with and without the quantitative report

Experience level	Rater#	No report	With report	Net change	*p* value
Consultant	Al	0.400	0.586	0.186	0.038*
	A2	0.469	0.571	0.102	
	A3	0.381	0.492	0.111	
Registrar	B1	0.455	0.211	− 0.244	0.68
	B2	0.522	0.571	0.05	
	B3	0.613	0.667	0.054	
Image analyst	Cl	0.169	0.531	0.362	0.66
	C2	0.746	0.556	− 0.19	
	C3	0.492	0.557	0.065	
Overall Mean (SD)		0.48 (0.17)	0.52 (0.13)	0.04	0.34

*Statistically significant at < 0.05

### Agreement and reliability across raters

For rating normal vs abnormal, Cronbach’s alpha for agreement across all raters showed improvement in overall rating reliability from 0.886 to 0.925 with the QReport available, corresponding to an improvement from ‘good’ to ‘excellent’. The intraclass correlation coefficient, assessed using mixed two-way ANOVA across raters, was 0.454 for single measures and 0.882 for average measures; with the QReport, these increased to 0.563 and 0.921, respectively.

### Power calculations

Based on our observed effect sizes of diagnostic accuracy (Table 2) for all raters, we have calculated the following sample size estimations to help inform future studies. To achieve an 80%, 90%, and 95% chance of observing a positive effect, 30, 40, and 45 raters would be required, respectively.

## Discussion

We performed a clinical accuracy study of our quantitative volumetric report (QReport—Fig. 1). Using an established segmentation algorithm, Geodesic Information Flows (GIF) [25], we developed a pipeline that brings together patient demographic information, hippocampal segmentation, brain parenchymal fraction, and global- and region-specific brain volumetry contextualised against a normative population (Fig. 1). The advantage of our ‘rose plot’ display is the representation of complex 3D data in a visually simple and easily interpretable 2D format. Our main aim was to assess the effect of our novel quantitative volumetric report on sensitivity, specificity, and accuracy across three neuroradiological levels of experience. Providing our QReport increased the sensitivity of detecting volume loss across all raters and improved the accuracy and agreement among the consultant group. It also improved sensitivity for diagnosing AD in the image analysts and for all raters combined, but had no effect on FTD discrimination. Further to this, the QReport reduced the variability in accuracy, sensitivity, and kappa scores for detecting volume loss. In absolute terms, the classification accuracy increased overall by over 5%. Given the documented increases in dementia prevalence in recent years and its future projections [32], this figure could be of clinical importance if confirmed in a larger study population.

Proprietary quantitative tools exist for the assessment of dementia, such as CorTechs.AI’s ‘Neuroquant’ (https://www.cortechs.ai/products/neuroquant/tbi/) and Icometrix’s ‘icobrain-dm’ (https://icometrix.com/products/icobrain-dm). Technical validation of their segmentation algorithms has been performed versus other segmentation procedures, with promising results [33, 34]. However, systematic assessment of their clinical accuracy by neuroradiologists, as addressed in the current study, has not been published for either, despite both tools being FDA and CE approved. Our ‘rose plot’ provides more intuitive information than numerical tables of sub-region volumes and limited visualisations of lobar and hippocampal volumes alone. There is a major lack of clinical validation studies in the literature for volumetric neuroradiological tools. In line with our research, a recent study showed improved identification of patients versus healthy controls for one of two raters, while both raters improved in the differential diagnosis of ‘dementing neurodegenerative disorders’ [21].

In a study using non-commercial algorithms, it was shown that adding lobar and hippocampal volumes to visual inspection improved the diagnostic accuracy of two experienced neuroradiologists [19], thereby mirroring our findings. This improvement suggests that experienced neuroradiologists are well placed to assimilate and make use of the information provided by the QReport. Furthermore, our consultant group showed the greatest statistical benefit due to having the least variance in their assessment performance between the two tasks, which is to be expected especially when compared with the non-clinical group (Table 2). Conversely, it is possible that less experienced neuroradiologists and non-clinical image analysts were over-reliant on the QReport for determining abnormality, as suggested by an overall decrease in specificity, although not significant (Tables 2 and 3).

When diagnosing a neurodegenerative disease on MR images, neuroradiologists first assess the presence of volume loss as well as its distribution. In a second step, they interpret the pattern to be indicative of a certain disease type, such as AD or FTD. In this context, it is worth noting that providing the QReport increased the sensitivity of the first step (the detection of volume loss across all raters) and improved the accuracy and agreement among the consultant group. For the differential diagnosis, the QReport improved sensitivity for AD in the image analysts and for all raters combined but had no effect on FTD. From a diagnostic point of view, providing an objective measure to reproducibly assess volume loss with a decreased interrater variability is crucial and could be used clinically in a number of neurodegenerative diseases. The limited effects on the differential diagnosis on FTD could be due to the low mean age of patients (61.7 years for AD and 59.9 years for FTD) and relatively short disease durations (2.7 years for AD and 3.5 years for FTD) (Table 1). This will have affected the degree of atrophy present and possibly made them harder cases to assess. However, it is also important to identify atrophy in younger patients while it is still subtle, and it is in these cases especially where a QReport could help reduce subjective visual disagreement.

**Table 6:** Kappa scores for agreement between rated diagnosis and clinically/CSF-confirmed AD and FTD diagnoses across all experience levels, both with and without the quantitative report

Experience Level	Rater#	No report	With report	Net change	*p* value
Consultant	Al	0.432	0.531	0.099	0.04*
	A2	0.45	0.498	0.048	
	A3	0.335	0.434	0.099	
Registrar	B1	0.381	0.22	− 0.161	0.56
	B2	0.326	0.428	0.102	
	B3	0.494	0.391	− 0.103	
Image analyst	Cl	0.02	0.176	0.156	0.28
	C2	0.529	0.496	− 0.033	
	C3	0.396	0.529	0.133	
Overall Mean (SD)		0.37 (0.15)	0.41 (0.13)	0.037	0.39

*Statistically significant at < 0.05

Interestingly, confidence in detecting volume loss and differentiating AD and FTD was not significantly affected by the QReport. Significantly increased confidence was unexpectedly shown when incorrectly diagnosing volume loss (i.e. false confidence) independent of experience level. One potential explanation is that raters based their incorrect diagnosis on visual inspection alone and used the QReport to reinforce their diagnosis. Irrespective of the reason, more work needs to be done to understand and mitigate this finding. It highlights the need for rigorous validation before clinical adoption and the importance of appropriate training to avoid over-reliance on diagnostic aids, completion of a test case set, and carefully planning and monitoring the introduction of tools such as the QReport. Rather than a gold standard, quantitative reports should be considered support tools which cannot replace neuroradiological experience, and raters should be wary of over-reliance.

### Limitations

Our study was somewhat limited in statistical power, due potentially to the subject sample size or the number of raters used. However, our sample size of 45 subjects was in line with other similar studies using between 36 and 52 subjects [17, 19, 20]. The use of nine raters within three experience levels enabled us to identify the effect of experience when introducing QReports. Similar work has used a total of 2 raters [19, 20] or a maximum of 3 raters [17]. The performance of our image analyst group was unexpectedly heterogeneous, likely due to disparity in experience level. The variability in the results within the image analysts and registrar groups could also reflect an over-reliance on the report, rather than using it in addition to the MRI. The ‘control’ group was half the size of the patient group, which could have contributed to unexpected decreases in specificity, although not significant (Tables 2 and 3). Our study therefore underlines the importance of considering sample sizes and rater groups when developing and validating such quantitative diagnostic aids. Future work will need to recruit more raters to better assess the effects of the report in diagnostic performance, and the moderators of this effect (see “Power calculations” in the “Results” section).

The ‘control’ subjects were recruited from a clinical population who all presented with subjective neurological complaints. It is possible that radiologically normal ‘controls’ had other pathologies, which may have affected our raters’ performance. This was, however, a conscious choice to reflect the clinical setting in memory clinics. Finally, the incidence ratio (Controls:AD:FTD), forced-choice nature, and lack of further clinical data in this study are not a reflection of routine neuroradiological assessment where more diagnostic options need to be considered.

### Conclusions

The results of this clinical accuracy study demonstrate that quantitative volume reports providing single-subject results referenced to normative data can improve the sensitivity, accuracy, and inter-observer agreement for detecting volume loss and AD. This is a crucial step when reporting volume changes in patients with dementia. The largest beneficial effect of the QReport was in the consultant group, suggesting they were best placed to assimilate and make use of the information provided by the QReport. The differing effects between all three experience levels highlight the need for studies clarifying the potential benefits and limitations of these reports, and the importance of rigorous validation before clinical adoption. Our sample sizes were low, but the effect sizes across accuracy and sensitivity were moderate-to-large in favour of a beneficial report effect. Importantly, a reduced variability in sensitivity, accuracy, and kappa scores was also noted. We believe our study will help to inform power calculations and study design for future research in the field.

#### Software availability

The software is non-commercial and a QReport can be freely generated by uploading a T1-weighted scan via this weblink—http://niftyweb.cs.ucl.ac.uk/program.php?p=QNID.

## Supplementary information

ESM 1 (DOCX 33 kb)

## Abbreviations

AD: Alzheimer’s disease
ADNI: Alzheimer’s Disease Neuroimaging Initiative
BPF: Brain parenchymal fraction
CAU: Caudate
CNR: Contrast-to-noise ratio
FTD: Frontotemporal dementia
GIF: Geodesic information flows
GM: Grey matter
QNI: Quantitative neuroradiology initiative
QReport: Quantitative volumetry report
SNR: Signal-to-noise ratio
WM: White matter
