# Supplementary material for: Automated quantitative MRI volumetry reports support diagnostic interpretation in dementia: a multi-rater, clinical accuracy study
Source: Eur Radiol. 2021 Jan 15;31(7):5312–23. doi: 10.1007/s00330-020-07455-8 (PMC8213665; doi:10.1007/s00330-020-07455-8)
Supplement: Supplementary file 1 — (DOCX 33 kb) [file 330_2020_7455_MOESM1_ESM.docx]

**Supplementary Material**

Acquisition parameters

Patient dataset – 41 scans on Trio MRI scanner, 3 scans on Skyra MRI scanner (TR=2,200 ms, TI=900 ms, TE=2.9 ms, acquisition matrix=256×256, FOV=26 cm, spatial resolution=1.1 mm) and 1 scan on Prisma MRI scanner (TR=2000 ms, TI=850 ms, TE=2.93 ms, acquisition matrix=256×256, FOV=26 cm, spatial resolution=1.1 mm).

Normative dataset – The ADNI-2 MRI protocol involved sagittal plane acquisition using an MP-RAGE/IR-FSPGR pulse sequence on a range of 3.0T MRI systems. High-resolution 3D T1-weighted images were acquired using an 8-channel coil, TR=400ms, TE=min full, flip-angle=11°, slice thickness=1.2 mm, resolution=256 × 256 mm and FOV=26 cm. In the TRACK-HD MRI protocol, the T1-weighted image volumes were acquired using a 3D MPRAGE acquisition sequence on 3.0T Siemens or a Phillips MRI system with the following imaging parameters: TR=2200ms (Siemens)/ 7.7ms (Philips), TE=2.2ms (S)/3.5ms (P), FOV=28cm (S)/ 24cm (P), matrix size 256x256(S)/224x224(P), 208(S)/164(P) sagittal slices to cover the entire brain with a slice thickness of 1.0 mm with no gap.

Instructions to raters

Please see below for verbatim instructions supplied to raters through the QNI website:

We have developed a tool to assist MRI interpretation for patients with suspected dementia. It is an automated grey matter (GM) segmentation technique which calculates whole brain and regional GM volumes from a subject's MRI scan, and presents a QNI report in the context of a normative range of control subjects.

We are keen to test how helpful it is for radiologists and other imaging staff to have this quantitative information. In this exercise, you will see a mixture of MRI scans from individuals with Alzheimer's dementia (AD), frontotemporal dementia (FTD), and those who are cognitively normal. We have chosen to focus on AD and FTD at present due to their distinctive patterns of atrophy.

You will see each scan in three planes and you will be able to scroll through the images as you would normally. You will see the same scans twice, once with and once without a QNI report, in a randomly mixed order. Please use the QNI report where it is available to assist you in making your judgement, otherwise please rely on your visual interpretation as you would normally.

An example QNI report appears on the right. The graph at the top indicates the subject's whole-brain volume. The 'bullseye' plot below shows the percentile of the GM volume in each lobe and important sub-regions, either higher (green) or lower (red) percentile.

For each scan, we will ask you to give your assessment:

1. Your overall impression – normal or abnormal
2. If you think the scan is abnormal, specify your diagnosis – AD or FTD
3. How confident you feel in your diagnosis on a scale 1-5, 1=not at all confident, 5=very confident.

Once you have finished looking at a scan, click **NEXT** and your assessment will be saved. To stop and come back to the exercise at any time, click on **MENU** in the top right hand corner of the screen. The next time you log in, the website will take you to the next scan to be reported. You can zoom in and out on the report using your mouse wheel.

Thank you again for your participation, we look forward to sharing the results with you!
